# COVID‐19: Current and future challenges in spine care and education ‐ a worldwide study

**DOI:** 10.1002/jsp2.1122

**Published:** 2020-08-28

**Authors:** Michael T. Nolte, Garrett K. Harada, Philip K. Louie, Michael H. McCarthy, Arash J. Sayari, G. Michael Mallow, Zakariah Siyaji, Niccole Germscheid, Jason PY Cheung, Marko H. Neva, Mohammad El‐Sharkawi, Marcelo Valacco, Daniel M. Sciubba, Norman B. Chutkan, Howard S. An, Dino Samartzis

**Affiliations:** ^1^ Department of Orthopaedic Surgery Rush University Medical Center Chicago Illinois USA; ^2^ The International Spine Research and Innovation Initiative RUSH University Medical Center Chicago USA; ^3^ Department of Orthopaedic Surgery Hospital for Special Surgery New York New York USA; ^4^ Research Department AO Spine International Davos Switzerland; ^5^ Department of Orthopaedics and Traumatology The University of Hong Kong Hong Kong; ^6^ Department of Orthopaedic and Trauma Surgery Tampere University Hospital Tampere Finland; ^7^ Department of Orthopaedic and Trauma Surgery Assiut University Medical School Assiut Egypt; ^8^ Department of Orthopaedics Churruca Hospital de Buenos Aires Buenos Aires Argentina; ^9^ Department of Neurosurgery John Hopkins University Baltimore Maryland USA; ^10^ Department of Orthopaedic Surgery University of Arizona College of Medicine Phoenix Arizona USA

**Keywords:** COVID‐19, coronavirus, education, future, guidelines, healthcare, impact, spine

## Abstract

**Background:**

The COVID‐19 pandemic has impacted spine care around the globe. Much uncertainty remains regarding the immediate and long‐term future of spine care and education in this COVID‐19 era.

**Study design:**

Cross‐sectional, international study of spine surgeons.

**Methods:**

A multi‐dimensional survey was distributed to spine surgeons around the world. A total of 73 questions were asked regarding demographics, COVID‐19 observations, personal impact, effect on education, adoption of telemedicine, and anticipated challenges moving forward. Multivariate analysis was performed to assess factors related to likelihood of future conference attendance, future online education, and changes in surgical indications.

**Results:**

A total of 902 spine surgeons from seven global regions completed the survey. Respondents reported a mean level of overall concern of 3.7 on a scale of one to five. 84.0% reported a decrease in clinical duties, and 67.0% reported a loss in personal income. The 82.5% reported being interested in continuing a high level of online education moving forward. Respondents who personally knew someone who tested positive for COVID‐19 were more likely to be unwilling to attend a medical conference 1 year from now (OR: 0.61, 95% CI: [0.39, 0.95], *P* = .029). The 20.0% reported they plan to pursue an increased degree of nonoperative measures prior to surgery 1 year from now, and respondents with a spouse at home (OR: 3.55, 95% CI: [1.14, 11.08], *P* = .029) or who spend a large percentage of their time teaching (OR: 1.45, 95% CI: [1.02, 2.07], *P* = .040) were more likely to adopt this practice.

**Conclusions:**

The COVID‐19 pandemic has had an adverse effect on surgeon teaching, clinical volume, and personal income. In the future, surgeons with family and those personally affected by COVID‐19 may be more willing to alter surgical indications and change education and conference plans. Anticipating these changes may help the spine community appropriately plan for future challenges.

## Graphical abstract

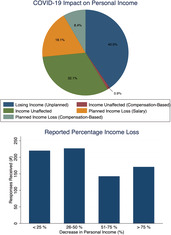

## 1 INTRODUCTION

The COVID‐19 pandemic has affected both patients and healthcare providers worldwide. At the time of this study's completion, inpatient hospital and intensive care unit demand have far exceeded capacity for many regions. Hospitals have made drastic alterations to care structures, including prioritizing different types of procedures and deferring elective surgeries.^1^, ^2^ In addition, many spine and orthopedic surgeons have been asked to help provide care outside of their area of expertise, including the emergency department and intensive care unit.^3^, ^4^ For many, the length of time that these changes will remain in place is unknown, and the pandemic may continue to unfold in stages.^4^, ^5^ The long‐term repercussions of the pandemic and its effects on healthcare providers are fraught with uncertainty.

As highlighted by Louie et al, spine surgeons around the globe have been affected by these aforementioned changes.^6^ Many have experienced diminished clinical productivity, adverse effects on personal income, and travel bans. In some cases, they have even been reassigned to work on the front lines.^3^, ^7^ Limitations from hospital systems have made it difficult for spine surgeons to effectively treat their patients. Many with myelopathic symptoms, worsening radicular pain, and motor weakness have been forced to struggle through continued conservative treatment.^8^, ^9^ An understanding of the challenges faced by spine surgeons in this current pandemic and their attitudes towards the future may help to guide resource allocation moving forward. Similarly, embracing positive changes that have resulted from this unique time may stimulate a new age of virtual learning and global collaboration. Thus, the aim of the present study was to elicit the attitudes, stresses, and future anticipations of spine surgeons around the globe as they pertain to patient care and education in response to the COVID‐19 pandemic, and to present tangible plans to address the challenges and concerns as they return to their clinical practices in the postCOVID‐19 era.

## 2 METHODS

### 2.1 Survey design and content

A survey, known as the AO Spine COVID‐19 and spine surgeon global impact survey, was developed. The survey panel was composed of five regional Research Chairs of AO Spine, representing seven global regions (Africa, Asia, Australia, Europe, Middle East, North America, and South America/Latin America), as well as long‐standing, experienced spine surgeons who have directed spine fellowship programs, epidemiologists, statisticians, and spine fellows as noted by Louie et al.^6^ Question selection was based on a Delphi style for consensus, following several rounds of review before finalization. The multiple‐choice questions included several domains, such as: demographics, COVID‐19 observations, financial impact, and future perceptions.

### 2.2 Survey distribution

The 73‐item survey was presented in English and distributed via email to the AO Spine membership who agreed to receive surveys (n = 3805). AO Spine is the world's largest society of international spine surgeons (www.aospine.org). The survey recipients were provided 9 days to complete the survey (27 March 2020 to 4 April 2020). Respondents were informed their participation was voluntary, and information gained would be disseminated publicly and in peer‐review journals.

### 2.3 Statistical analysis

All statistical analysis and data processing were performed using open‐source packages through the Python v3.7 programming language. Survey responses were summarized via collection of raw count data and respective calculation of percentages, means, and standard deviations. Multivariate logistic regression analysis was performed to assess variations in survey responses focusing on patient care/education and included reported interest level in online spine education, likelihood of attending a medical conference in 1 year, and inclination to use increased nonoperative measures prior to surgery in 1 year. Model covariates believed to be relevant to dependent variables of interest based on the opinions of senior co‐authors, and included all collected baseline survey respondent demographics and practice‐specific characteristics. Results of the multivariate models were summarized via calculation of odds ratios (ORs), 95% confidence intervals (CIs), and *P*‐values. A *P*‐value <.05 was used to establish statistical significance for all tests.

## 3 RESULTS

**FIGURE 1:**
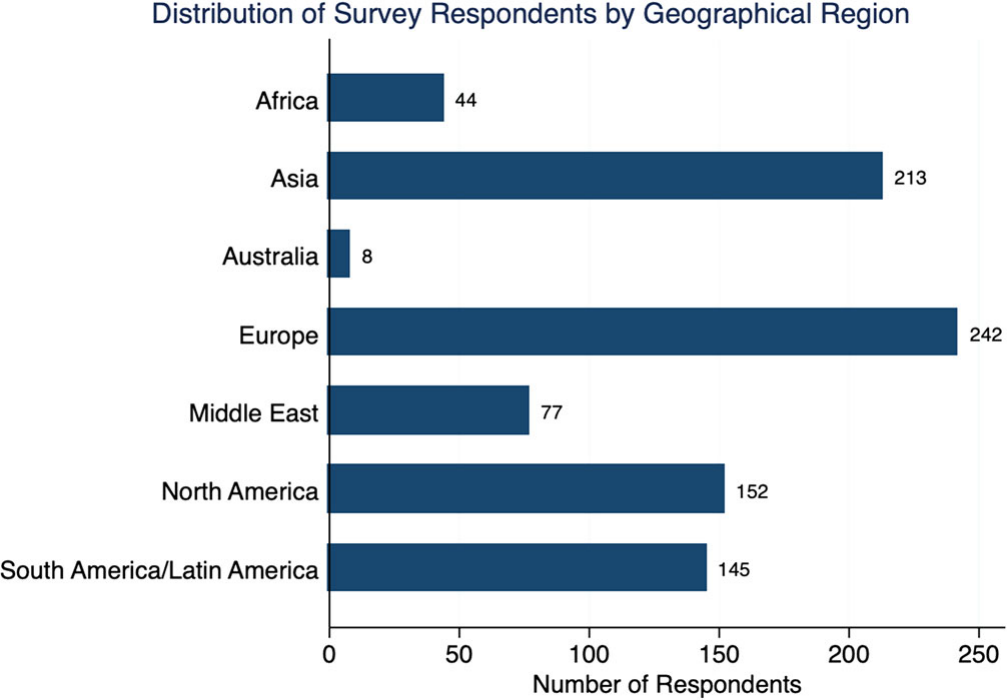
Distribution of survey respondents by geographical region

In total, 902 surgeons completed the survey, with an overall response rate of 23.7%. Of the 7 global regions represented, those with the greatest number of responses were Europe (242/881; 27.5%), followed by Asia (213/881; 24.2%) and North America (152/881; 17.3%). Of 91 countries, the majority of survey responses were from the United States (128/902; 14.2%), China (73/902; 8.1%), and Egypt (66/902; 7.3%) **(**Figure 1 **).** More respondents were male (826/881; 93.8%) orthopedic surgeons (637/902; 70.6%), aged 35‐44 years‐old (344/895; 38.4%), and primarily practiced in academic institutions (405/892; 45.4%). Of note, a majority reported having a spouse at home (773/902; 86.5%) and children at home (637/887; 71.8%) **(**Table 1 **).**

**TABLE 1:** Survey respondent demographics

Personal demographics			Practice demographics		
	#	%		#	%
Age (Y)			Specialty		
25‐34	130	14.5	Orthopedics	637	70.6
35–44	344	38.4	Neurosurgery	246	27.3
45‐54	245	27.4	Trauma	104	11.5
55‐64	150	16.8	Pediatric surgery	17	1.9
65+	26	2.9	Other	35	3.9
Sex			Years since training completion		
Female	55	6.2	Less than 5 Y	161	25.3
Male	826	93.8	5 to 10 Y	141	22.2
Home demographics			10 to 15 Y	104	16.4
Spouse at home	773	86.5	15 to 20 Y	117	18.4
Children at home			Over 20 Y	113	17.8
0	250	28.2	Practice Type		
1	221	24.9	Academic/Private Combined	204	22.9
2	266	30.0	Academic	405	45.4
3	109	12.3	Private	144	16.1
4+	41	4.6	Public/Local Hospital	139	15.6
Elderly at home	191	21.4	Practice Breakdown (%)		
Living alone	63	7.1	% Research		
Geographic region			0–25	731	81.9
Africa	44	5.0	26‐50	129	14.5
Asia	213	24.2	51‐75	21	2.4
Australia	8	0.9	76‐100	12	1.3
Europe	242	27.5	% Clinical		
Middle East	77	8.7	0–25	22	2.5
North America	152	17.3	26–50	87	9.7
South America/Latin America	145	16.5	51–75	194	21.7
Exposure to previous pandemics			76–100	590	66.1
SARS	98	47.1	% Teaching		
H1N1 (Swine Flu)	128	61.5	0–25	668	74.9
MERS	17	8.2	26–50	152	17.0
Ebola	15	7.2	51–75	50	5.6
Total respondents	902	100	76–100	22	2.5

Abbreviations: #, number of respondents/votes, %, percent, SARS, severe acute respiratory syndrome; MERS, Middle East respiratory syndrome.

Respondents reported a moderate‐ to high‐level of overall concern regarding the COVID‐19 outbreak, with a mean score of 3.7 on a scale of one to five. The three most common stressors identified were family health (76.0%), economic concerns (45.7%), and timeline to resume regular clinical practice (44.9%). Hospitals did not have guidelines for dealing with an outbreak for 39.6% of respondents, and 94.7% said that formal guidelines should be developed moving forward.

**FIGURE 2:**
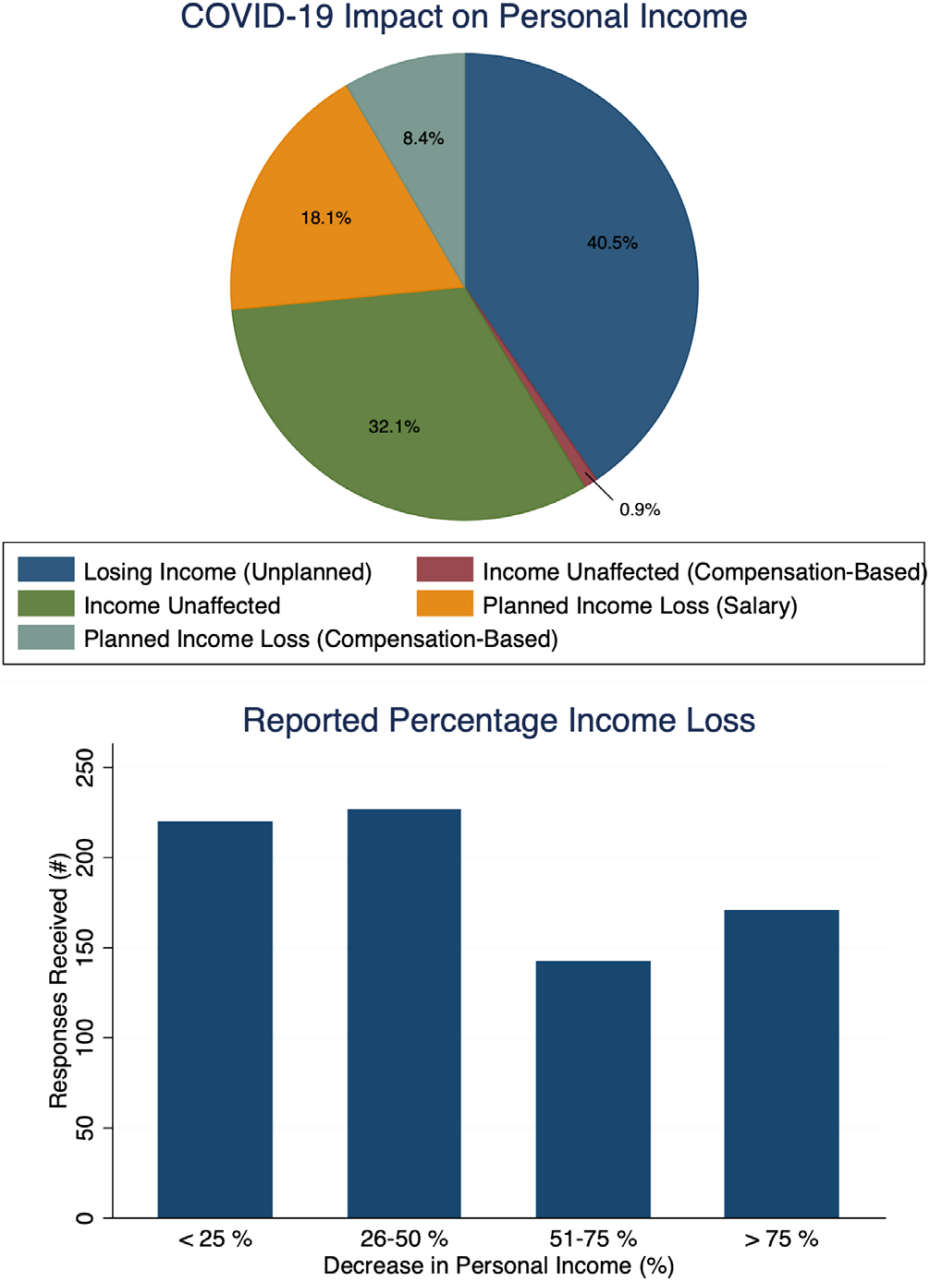
Impact of COVID‐19 pandemic on personal income loss

Furthermore, these concerns matched reported changes in daily clinical practice, income, and anticipations to resume normal elective surgical activity. Time spent performing clinical duties had decreased for 84.0%. At the time of survey completion, 100% of respondents reported that their surgical volume was adversely affected, and 67.1% of respondents stated that over 75.0% of their surgical caseload had been either postponed or canceled. Financially, 67.0% of surgeons reported an expected loss of income **(**Figure 2), and a majority reported an expected decrease in revenue both personally and for their employer at the time of survey completion. In addition, 48.8% of respondents stated that the timeframe to resume elective surgeries is unknown. Assuming normal operative permission for elective surgery, 64.0% anticipated more than 4 weeks and 26.5% anticipated more than 8 weeks before a return to “baseline” status is possible.

There is also concern that the presence of the virus may be felt for years to come. When asked how this outbreak will impact patient care 1 year from now, 57.9% said they will have a heightened awareness of hygiene, and 45.8% will increase use of personal protection equipment. Providers may be more hesitant to offer surgical intervention going forward, as 20.0% reported they plan to pursue an increased degree of nonoperative measures prior to surgery and 38.0% will have patients reschedule if they feel sick. Furthermore, when asked if they would attend a scheduled medical conference 1 year from now, 33.7% reported being either unsure or not likely.

Multivariate assessment of anticipated clinical practice changes revealed that respondents are more inclined to pursue nonoperative measures in 1 year if he/she reported having a spouse at home (OR: 3.55, 95% CI: [1.14, 11.08], *P* = .029) or spent a large percentage of their time teaching (OR:1.45, 95% CI: [1.02, 2.07], *P* = .040). Conversely, orthopedic respondents (OR: 0.17, 95% CI: [0.01, 0.65], *P* = .009), North American clinicians (OR: 0.25, 95% CI: [0.01, 0.94], *P* = .040), and those who did not expect the COVID‐19 pandemic to impact their salary (OR: 0.39, 95% CI: [0.19, 0.81], *P* = .011) were less likely to implement future nonoperative management (Table 2).

**TABLE 2:** Inclination to pursue nonoperative measures in 1 year

Survey questions	Odds ratio	95% Confidence interval	*P*‐value^a^
Demographics			
Sex	0.46	(0.15, 1.44)	.184
Age	1.15	(0.74, 1.77)	.534
Number of Children at Home	0.92	(0.72, 1.17)	.478
Spouse at home	3.55	(1.14, 11.08)	**.029**
Region			
Asia	1.66	(0.52, 5.32)	.393
Australia	0.30	(0.02, 3.79)	.355
Europe	0.93	(0.28, 3.05)	.901
Middle East	0.83	(0.22, 3.19)	.783
North America	0.25	(0.07, 0.94)	**.040**
South/Latin America	0.64	(0.19, 2.19)	.473
Specialty			
Orthopedics	0.17	(0.05, 0.65)	**.009**
Neurosurgery	0.24	(0.06, 0.92)	**.038**
Trauma	0.80	(0.30, 2.10)	.649
Pediatrics	0.00	(<0.00, 1.25 × 10^235^)	.966
Other	1.14	(0.30, 4.36)	.846
Income impact			
No Impact: Compensation‐based income	4.42	(0.46, 42.33)	.198
No impact: Salary	0.39	(0.19, 0.81)	**.011**
Reduction: Salary	0.97	(0.49, 1.89)	.918
Reduction: Compensation‐based income	0.90	(0.35, 2.31)	.832
Practice time allocation			
Clinic	0.92	(0.64, 1.32)	.633
Research	1.21	(0.73, 2.00)	.460
Teaching	1.45	(1.02, 2.07)	**.040**
Previous outbreak			
SARS	0.81	(0.36, 1.80)	.599
H1N1 (Swine Flu)	1.04	(0.49, 2.23)	.911
MERS	3.12	(0.60, 16.20)	.175
Ebola	1.63	(0.25, 10.81)	.612
Recommend precautions for spine surgery			
Recommend against	1.65	(0.92, 2.98)	.096
Standard level of caution	1.00	(0.50, 1.97)	.991
Not present during intubation/extubation	0.86	(0.45, 1.65)	.653
Addition PPE during surgery	1.71	(0.92, 3.17)	.089
Other			
Practice type	0.93	(0.73, 1.18)	.528
Years since training completion	0.90	(0.67, 1.20)	.453
Clinical duties impacted	1.09	(0.70, 1.70)	.718
Personal revenue loss expected	0.98	(0.75, 1.28)	.868
Currently performing elective cases	0.66	(0.33, 1.32)	.242

^a^ *P*‐values were calculated using multivariate logistic regression analysis. Bolded values indicate statistical significance at *P* < .05.

Abbreviations: SARS, severe acute respiratory syndrome, MERS, Middle East respiratory syndrome.

Scholarly activity and in‐person education has been adversely affected, with 66.3% reporting that their research activities had decreased in productivity or completely stopped. Similarly, of respondents with residents and fellows, 85.2% reported that the pandemic has hurt their overall experience. However, there has been a stimulation of telemedicine, web‐based learning, and global collaboration in response to the pandemic. One year from now, 41.8% of respondents anticipated having an increased use of digital options for patient communication. Regarding online spine education, 82.5% reported being either interested or very interested in participating and continuing these efforts. If a centralized web‐based collaboration platform were established, 33.9% said they would be active readers, and 59.8% said they would both read and contribute.

Multivariate analysis of present and future academic activity also demonstrated significant associations with various responses. Clinicians being government mandated to shelter in place (OR: 2.59, CI: [1.14, 5.87], *P* = .022) or those with practices previously impacted by a prior pandemic (SARS) (OR: 2.49, CI: [1.30, 4.76], *P* = .006) were significantly more likely to attend medical conferences in 1 year. In contrast, those unlikely to attend reported knowing someone who has tested positive for COVID‐19 (OR: 0.61, CI: [0.39, 0.95], *P* = .029) (Table 3). Lastly, surgeons reporting larger clinic‐based practices were more likely to express interest in online spine education (OR: 2.56, CI: [1.07, 6.12], *P* = .034) (Table 4).

**TABLE 3:** Likelihood of attending a conference in 1 year

Survey questions	Odds ratio	95% Confidence interval	*P*‐value^a^
Demographics			
Region	0.96	(0.85, 1.09)	.543
Sex	0.93	(0.30, 2.90)	.899
Age	0.92	(0.71, 1.19)	.518
Children	1.02	(0.83, 1.25)	.870
Spouse at home	1.85	(0.79, 4.35)	.156
Previous Outbreak			
SARS	2.48	(1.30, 4.76)	**.006**
H1N1 (Swine Flu)	0.50	(0.24, 1.01)	.054
MERS	1.41	(0.30, 6.51)	.662
Ebola	0.63	(0.11, 3.55)	.598
Specialty			
Orthopedics	1.02	(0.26, 4.06)	.975
Neurosurgery	0.89	(0.22, 3.57)	.871
Trauma	0.56	(0.22, 1.43)	.224
Pediatrics	1.52	(0.21, 10.97)	.678
Other	1.10	(0.29, 4.13)	.891
Practice time allocation			
Clinic	0.95	(0.68, 1.33)	.756
Research	1.41	(0.90, 2.21)	.139
Teaching	0.94	(0.69, 1.29)	.720
Other			
Practice type	0.85	(0.66, 1.09)	.195
Years since training completion	0.98	(0.83, 1.15)	.767
Personal exposure to COVID‐19	0.61	(0.39, 0.95)	**.029**
Clinical duties impacted	1.40	(1.00, 1.96)	**.049**
Government mandate to shelter	2.59	(1.14, 5.88)	**.022**
Income impacted	0.91	(0.78, 1.07)	.241
Personal revenue loss expected	0.87	(0.71, 1.05)	.145
Hospital staff furloughed	1.03	(0.71, 1.50)	.876
Negative research impact	0.91	(0.77, 1.08)	.259

^a^ *P*‐values were calculated using multivariate logistic regression analysis. Bolded values indicate statistical significance at *P* < .05.

Abbreviations: SARS, severe acute respiratory syndrome, MERS, Middle East respiratory syndrome.

**TABLE 4:** Interest in Participating in online spine education

Survey questions	Odds ratio	95% Confidence interval^b^	*P*‐value^a^
Demographics			
Sex	1.00	(0.07, 15.42)	1.000
Age	1.60	(0.51, 4.98)	.420
Number of children at home	0.64	(2.00, 1.10)	.106
Spouse at home	1.28	(0.17, 9.88)	.811
Region			
Asia	6.07	(0.63, 58.61)	.119
Australia	2.430 × 10^3^	(0, inf)	.938
Europe	2.42	(0.28, 20.83)	.421
Middle East	1.27	(0, inf)	.997
North America	2.85	(0.33, 24.72)	.341
South/Latin America	3.31	(0.38, 28.99)	.280
Specialty			
Orthopedics	2.66	(0.01, 5.32)	.386
Neurosurgery	4.43	(0.03, 7.67)	.576
Trauma	5.69	(0, inf)	.998
Pediatrics	6.05	(0, inf)	.950
Other	779.61	(0, inf)	.936
Perceived impact on resident training			
Negative	3.67	(0.98, 13.69)	.053
None	1.18	(0.19, 7.35)	.861
Positive	4.49 × 10^7^	(0, inf)	.997
Practice time allocation			
Clinic	2.56	(1.07, 6.12)	**.034**
Research	6.83 × 10^9^	(0, inf)	1.000
Teaching	3.54	(0, inf)	.227
Previous outbreak			
SARS	0.84	(0.13, 5.50)	.854
H1N1	1.04	(0.10, 2.45)	.377
MERS	6.05 × 10^4^	(0, inf)	.969
Ebola	6.16 × 10^3^	(0, inf)	.950
Other			
Government mandate to shelter	1.33	(0.46, 6.44)	.417
Inclination to pursue nonoperative measures in 1 y	0.48	(0.28, 24.87)	.398
Negative research impact	1.04	(0.67, 1.61)	.852
Percentage of clinic performed via telecommunication	0.97	(0.631, 1.65)	.934
Practice type	1.82	(0.44, 1.13)	.144
Number years since posttraining	0.79	(0.40, 1.54)	.478

Abbreviations: SARS, severe acute respiratory syndrome, MERS, Middle East respiratory syndrome.

^a^ *P*‐values were calculated using multinominal logistic regression analysis. Bolded values indicate statistical significance at *P* < .05.

^b^ 95% Confidence intervals with an upper bound of infinity indicates a failure of convergence, whereby an estimated range for a given odds ratio could not be calculated.

## 4 DISCUSSION

The findings of the present study suggest that spine surgeon's worldwide share in the concern surrounding the COVID‐19 pandemic. Clinical volume has diminished, daily routines have been altered, and revenues have decreased. There is uncertainty as to what both the immediate and distant future holds. Despite these concerns, there has been a renewed interest in telemedicine, virtual learning, and global collaboration. Guidelines are necessary to help spine surgeons meet the challenges of current and future patient care, in addition to maintaining efforts aimed at education and collaboration **(**Table 5 **)**.

**TABLE 5:** Anticipated future challenges and tips for spine surgeons

Immediate challenges	Triage surgical pathology from urgent to elective Discuss and document surgical indications with colleagues Utilize appropriate personal protective equipment and protocols Consider adopting telemedicine services
Future challenges	Collaborate and help establish hospital guidelines for future pandemics or similar events Maintain financial flexibility and utilize government resources where appropriate
Education and collaboration	Continue use of virtual learning formats that have been established during the pandemic Attend meetings that have transitioned to online environments Collaborate through global spine communication and education platforms

### 4.1 Spine care in the future

In regards to spine care moving forward, roughly one in five surgeons reported that they plan to attempt longer courses of conservative care before committing to surgical intervention 1 year from now. This may be reflective of surgeons’ hesitancy to bring patients to the hospital, exposing both patients and care teams to the potential for pathogen transmission. A more likely explanation, however, may be an effort to avoid overwhelming a stressed healthcare system with unplanned hospital or ICU admissions.^10^ This practice of attempting a longer course of conservative therapy was more likely to be adopted by those with a spouse at home, likely reflective of an overarching desire to avoid spreading COVID‐19 to the home environment.^11^, ^12^ Interestingly, this practice was also more likely to be adopted by clinicians who identified as spending a large portion of their time in resident and fellow education, and less likely to be adopted by those who did not expect the pandemic to impact their salary. In addition to minimizing exposure of residents and fellows to COVID‐19, this finding is consistent with the higher likelihood of providers in academic settings receiving a fixed‐salary rather than reimbursement based solely on surgical volume.^13^ Regardless, forced consideration of elective surgeries moving forward may help providers to better identify patients that can be treated sufficiently with nonoperative care.^9^, ^14^ Once hospital capacity for performing spine surgeries does return to normal or near‐normal, we encourage spine surgeons to maintain evidence‐based indications for conservative care vs surgical intervention. The practice of peer‐to‐peer communication and indications review regarding the appropriateness of treatment plans, both nonoperative and operative should be continued into the distant future to ensure an appropriate treatment plan and allocation of resources.

The COVID‐19 pandemic has been accompanied by considerable financial uncertainty for spine surgeons. In fact, the timeline to resume clinical practice and economic concerns were identified as two of the three most common stressors shared by respondents to our survey. Furthermore, a majority reported experiencing a personal decrease in revenue, and half of respondents had no timeline for return to “normal” clinical care. This financial uncertainty for both practices and individual providers is not only a key source of stress, but also an issue that may carry lasting repercussions. Vaccaro et al shared the experience and tentative financial plan of three prominent orthopedic surgery practices with academic ties in the United States.^15^ All three reported an expected loss in revenue and income for the practice and care providers that last until the third quarter of the 2020 fiscal year, assuming that elective surgeries are able to restart in the summer of 2020. Each had enacted work hour restrictions or furlough for ancillary staff. The timeline for these changes remains unclear, and is dependent largely on the easing of surgery restrictions. Furthermore, when spine surgeons are able to perform elective procedures once again, their patient population may be considerably different from the prepandemic population. That is, patients may not have the same health insurance that they previously carried, patients may not have time to seek elective surgery following an extended period of diminished or no income, and many will simply not wish to seek care in a hospital setting where COVID‐19 is present.^16^ Although there have been some efforts in the United States to help provide fiscal support to struggling physicians and practices, such as the Coronavirus Aid, Relief and Economic Security (CARES) Act, the effectiveness of these interventions has yet to be seen.^17^ Spine surgeons should optimize financial and scheduling flexibility until a prepandemic level of productivity and fiscal security can be realized.

Finally, many respondents to our survey anticipate a long‐term emphasis on efforts to avoid a similar public health crisis in the future. When asked if they had any words they wished to share, a large proportion offered messages of caution and safety, with the largest percentage directed towards patients in particular. Similarly, the most likely changes that surgeons anticipated 1 year from now included an increased emphasis on hygiene and proper use of personal protective equipment. Habits such as these are critical, as multiple rebounds in the number of patients with COVID‐19 are expected until a vaccine becomes available or herd immunity can be realized.^18^ In addition, despite the fact that a minority of surgeons reported working at an institution that had guidelines for the pandemic, nearly all respondents believed that such guidelines should be a priority and in place moving forward. Clearly there is a need for evidence‐based protocols. Hospitals and medical centers must disclose anticipated needs and provide solutions to local government, at which point data can be gathered from multiple centers within a region. Plans can be developed at the local, regional, and national levels around the world to provide formal guidelines that are evidence‐based and aligned with the research and experience of the COVID‐19 pandemic.

### 4.2 Education and collaboration

The COVID‐19 pandemic has resulted in new sources of stress, changes to patient care, and uncertainty regarding future practice. The education of residents and fellows, in addition to the continued learning of faculty, has not been immune to these changes^1^, ^19^ A majority of respondents in our survey noted that the education of their residents and fellows had been adversely affected, and their personal research productivity had fallen or stopped. Despite many negatives, the pandemic has stimulated a wave of web‐based learning and global collaboration that has not been seen before.^20^ In fact, over 80% of respondents indicated being interested or very interested in the continued use of novel learning modalities. Interestingly, providers who reported spending a majority of their time in clinical medicine, vs teaching or research were more likely to express interest in this continued use. This may be attributable to the relative ease and convenience of participating and/or leading educational experiences on virtual platforms that simultaneously allow for a high‐volume clinical practice. Furthermore, this may be a valuable avenue for academic departments and professional societies and to engage spine providers in educational endeavors who were previously too occupied.

Virtual meeting platforms in particular have revolutionized the way clinicians interact and learn while spending time away from their normal work environment.^21^ The examples are numerous. Residency and fellowship programs in orthopedic surgery and neurosurgery have adopted online platforms for departmental conferences and meetings. Governing bodies and subspecialty societies within spine surgery have organized and led international seminars that surgeons can access from their own home. Industry and independent education foundations have held virtual case reviews, research study group meetings, and topical debates. In response to high attendance and positive feedback, time and money invested for these endeavors has risen drastically.^22^ Given the number of spine care providers that have met the learning curve and successfully adopted these platforms, our hope is that these powerful learning tools continue to serve important roles into the distant future.

Concerns regarding the spread of disease and placing spine care providers at risk has resulted in the cancellation or postponement of conferences around the world. Furthermore, once providers return to clinical practice, they may lack the financial and scheduling freedom to attend traditional in‐person conferences. Many of these conferences have therefore adopted virtual formats, in which presentations and resources can be accessed via computer remotely. However, a timeline to return to normalcy and a long‐term plan for medical conferences remains unknown. When asked if they would attend a scheduled conference 1 year from now, roughly two out of three respondents indicated that they would feel comfortable doing so. Those who reside in a region that had a shelter in place order, and those who reported being impacted by a prior pandemic (SARS), expressed a higher likelihood of feeling comfortable. This is likely reflective of provider confidence in local governing bodies to develop policies to limit the spread of a pathogen, recover once the pandemic has resolved, and to determine if and when a medical conference is appropriate.^23^, ^24^

Although face‐to‐face interaction is valuable, there has been a growing interest for global collaborative platforms for spine providers that may serve a similar function to medical conferences of the past. When asked in our survey, 34% reported they would be active readers on such a platform, and 60% reported they would both read and contribute. Spine governing bodies and subspecialty societies should consider these data when planning meetings and collaborative opportunities in a postCOVID‐19 world.

## 5 CONCLUSIONS

The effects of the COVID‐19 pandemic have been far reaching, many of which have been felt by spine surgeons around the world. The present study suggests that spine surgeons share a high level of concern regarding the pandemic, with drastic changes to daily routines, finances, and methods of learning. Understanding the current attitudes, sources of stress, and unique areas of interest for spine surgeons may enable planning and anticipation for future challenges. Web‐based learning and global collaboration have seen a unique surge in utilization. Rising to meet the challenges of COVID‐19 and maintaining the unanticipated benefits will help the field of spine surgery to continue moving forward.

## CONFLICT OF INTEREST

The authors declare no potential conflict of interest.

## AUTHOR CONTRIBUTIONS

All authors played an important role in the development of this manuscript. Michael T. Nolte, Arash J. Sayari, Dino Samartzis drafted and revised the manuscript. Garrett K. Harada, Philip K. Louie, Michael H. McCarthy, G. Michael Mallow, Zakariah Siyaji, Niccole Ger, and Dino Samartzis were responsible for data acquisition, analysis, and interpretation. Jason PY Cheung, Marko H. Neva, Mohammad El‐Sharkawi, Marcelo Valacco, Daniel M. Sciubba, Norman B. Chutken, Howard S. An, and Dino Samartzis were responsible for manuscript revision, and approval of final version. All authors have read and approved the final submitted manuscript.
